# Superflux of an organic adlayer towards its local reactive immobilization

**DOI:** 10.1038/s42004-023-01020-2

**Published:** 2023-10-18

**Authors:** David Salamon, Kristýna Bukvišová, Vít Jan, Michal Potoček, Jan Čechal

**Affiliations:** 1 CEITEC - Central European Institute of Technology, Brno University of Technology, Purkyňova 123, 612 00 Brno, Czech Republic; 2 Fakulty of Mechanical Engineering, Brno University of Technology, Technická 2896/2, 616 69 Brno, Czech Republic

**Keywords:** Surface assembly, Surfaces, interfaces and thin films

## Abstract

On-surface mass transport is the key process determining the kinetics and dynamics of on-surface reactions, including the formation of nanostructures, catalysis, or surface cleaning. Volatile organic compounds (VOC) localized on a majority of surfaces dramatically change their properties and act as reactants in many surface reactions. However, the fundamental question “How far and how fast can the molecules travel on the surface to react?” remains open. Here we show that isoprene, the natural VOC, can travel ~1 μm s^−1^, i.e., centimeters per day, quickly filling low-concentration areas if they become locally depleted. We show that VOC have high surface adhesion on ceramic surfaces and simultaneously high mobility providing a steady flow of resource material for focused electron beam synthesis, which is applicable also on rough or porous surfaces. Our work established the mass transport of reactants on solid surfaces and explored a route for nanofabrication using the natural VOC layer.

## Abstract (web summary)

Volatile organic compounds are found on a majority of surfaces where they change surface properties and act as reactants, however, their diffusion flow rates remain undetermined. Here, the authors show that isoprene travels approximately 1 μm/s on zirconia under ultrahigh vacuum and is polymerized by an electron beam.

## Introduction

On-surface mass transport is the key process determining the kinetics and dynamics of on-surface reactions, including the formation of nanostructures, catalysis, or surface cleaning^1–4^. Volatile organic compounds (VOC) localized on a majority of surfaces greatly impact material properties^5,6^, complicate the correct material characterization^7,8^, should be considered when designing fabrication protocols^9^, and are an important class of compounds affecting human health within indoor environments^10–12^. The presence of VOC is typically referred to as contamination that impacts the performance of manufactured semiconductor devices^11–13^, alters metal work function^6^ and subsequent energy level alignment with organic semiconductors^5,14,15^, and other surface properties of metals or ceramics like adhesion, surface free energy, hydrophobicity and biocompatibility^16–18^. Removal of the VOC is easily possible by plasma or thermal treatments; however, within several minutes to hours, an organic layer is present again on common inorganic surfaces^19^.

Mobility of the VOC is widely discussed, but the discussion is focused on adsorption and transport by air^20,21^. On the other hand, the presence of VOC is reported as a source of contamination during electron microscopies (SEM, TEM) when transport by the atmosphere is restricted^22^. There, the contamination is visible in areas exposed to the e-beam as the beam changes the chemical composition of VOC^23^. The capability of the e-beam to induce chemical reactions is widely employed for the fabrication of nanostructures from organic precursors either by crosslinking or breaking polymer chains in electron e-beam lithography^24^ or precursor decomposition in focused electron-beam-induced deposition (FEBID)^25,26^. The electrons with a low energy of 2.5–100 eV can induce crosslinking of self-assembled monolayers via electron impact ionization and dissociative electron attachment mechanisms^27^. Significant changes also occur on surfaces of materials without intentional exposure to resist films or reactive gas^28^ due to the presence of native carbon contaminations on surfaces of most common inorganic materials, which can be traced back to VOC origin^6^. The polymeric layers formed of surfaces exposed by electrons^28,29^ lower contrast, enhance electron attenuation in electron spectroscopies, and largely change the properties of surface areas measured after focused beam milling/deposition was performed^7,8,30,31^.

Indoor environments have surface area-to-volume ratios hundreds of times higher than outdoor environments^11,32^. The high surface-to-volume ratio means that surface chemistry impacts indoor air as surfaces adsorb and emit organic molecules and provide the environment for various chemical reactions to take place^10–12,32^. The thermodynamic and kinetic parameters of VOC adsorption are important inputs for models that include surface physical and chemical processes in the form of deposition and subsequent removal of compounds or organic film growth^10,32^. Mass transport of the adsorbed species is an indispensable part of these models because, especially at low partial pressures of the reactants, surface diffusion is the key process determining the kinetics and dynamics of on-surface reactions^1–4^. The significance of surface diffusion in electron beam transformation of adsorbed organic molecules was established already few decades ago^33^. Since then, the electron beam writing process has been investigated^28,29,31,34–38^, and the essential role of surface diffusion has been confirmed^28,29,37,38^. In the related field of FEBID, in which the precursor molecules are injected on purpose, the surface diffusion of precursors can significantly enhance the growth rates by replenishing the consumed monomers^26^. Reliable diffusion coefficients are necessary for a quantitative description of surface diffusion. While these are better established on metal surfaces, the quantitative information on diffusion coefficients is rather scarce on oxides.

Here we provide a quantitative answer to the fundamental question: “How far and how fast can the molecules travel on the surface to react?” We have determined the diffusion flow rates of prototypical VOC, isoprene (2-methyl-1,3-butadiene)^39^, on the zirconium oxide surfaces. We show that the flow provides a steady supply of reactants for spatially localized on-surface reaction, in our instance, electron beam (e-beam in following) induced polymerization. Our results indicate that the reaction taking place within a 20-nm electron beam gathers the material from a much larger area with a radius of several micrometers.

Surface diffusion from the surrounding area to the area under the electron beam is a thermally activated process; the probability of transfer of molecule to neighboring adsorption state (jump) can be described by diffusion coefficient $D={D}_{0}\exp (-{E}_{{{{{{\rm{sd}}}}}}}/{kT})$, where ${D}_{0}$ is a pre-exponential factor, ${E}_{{{{{{\rm{sd}}}}}}}$ the activation energy of surface diffusion, $k$ Boltzmann constant, and $T$ temperature^40^; ${D}_{0}$ and ${E}_{{{{{{\rm{sd}}}}}}}$ can be obtained by transition state theory^41^. For atomic species, the *E*_sd_ is typically 5–20% of activation energy for desorption *E*_des_^42^. However, for astrochemically significant molecules on water ice, values 20–70% of *E*_des_ were found with no apparent relation with desorption energies^1^. Alongside, surface science studies indicate that values of pre-exponential factors also depart significantly from the often-used universal value of 1 × 10^−3^ cm^2^s^−1^ at 300 K^43^. These discrepancies are associated with significant entropic contributions in the complex transition state of organic molecules with complicated internal (vibrational and rotational) degrees of freedom^43–45^. In addition, the surface diffusion shows significant concentration dependence with intricate cooperative effects^46–48^, highlighting the importance of studies beyond single molecule diffusion studies on monocrystalline surfaces by scanning tunneling microscopy^49^.

Here, we present a pilot study on the determination of the surface flow of isoprene, synthetic VOC in ultrahigh and high vacuum conditions on the ceramic surface of zirconia (tetragonal zirconia stabilized with 3 mol.% of Y_2_O_3_) and titania (TiO_2_). If not stated otherwise, all substrates were fully sintered with a density over 99.9 % of the theoretical value; thus, they do not contain a significant amount of open pores that could act as storage for organic material and interfere with the diffusion experiments. The isoprene organic adlayer was deposited by drop casting on the surfaces thermally cleaned (600 °C, 2 h). Afterward, the sample was introduced to a vacuum environment through a load-lock chamber, where the majority of isoprene evaporated, leaving a thin organic film on the surface. We have minimized the influence of further adsorption by performing the studies in ultrahigh vacuum (10^-7 ^Pa) conditions. We challenge the common thinking of contamination and present the huge opportunity we recognize in the utilization of VOC for the growth of functional nanostructures by e-beam-induced polymerization^50^ going beyond the recent strategies for VOC immobilization^20,32^. Our study reveals that it is possible to 3D print polymeric nanostructures on rough, non-planar, non-conductive ceramic surfaces surpassing thus the requirement of a smooth planar surface for the photoresist application required for EBL.

## Results

### Superflux of VOC on the ceramic surface

**Fig. 1:**
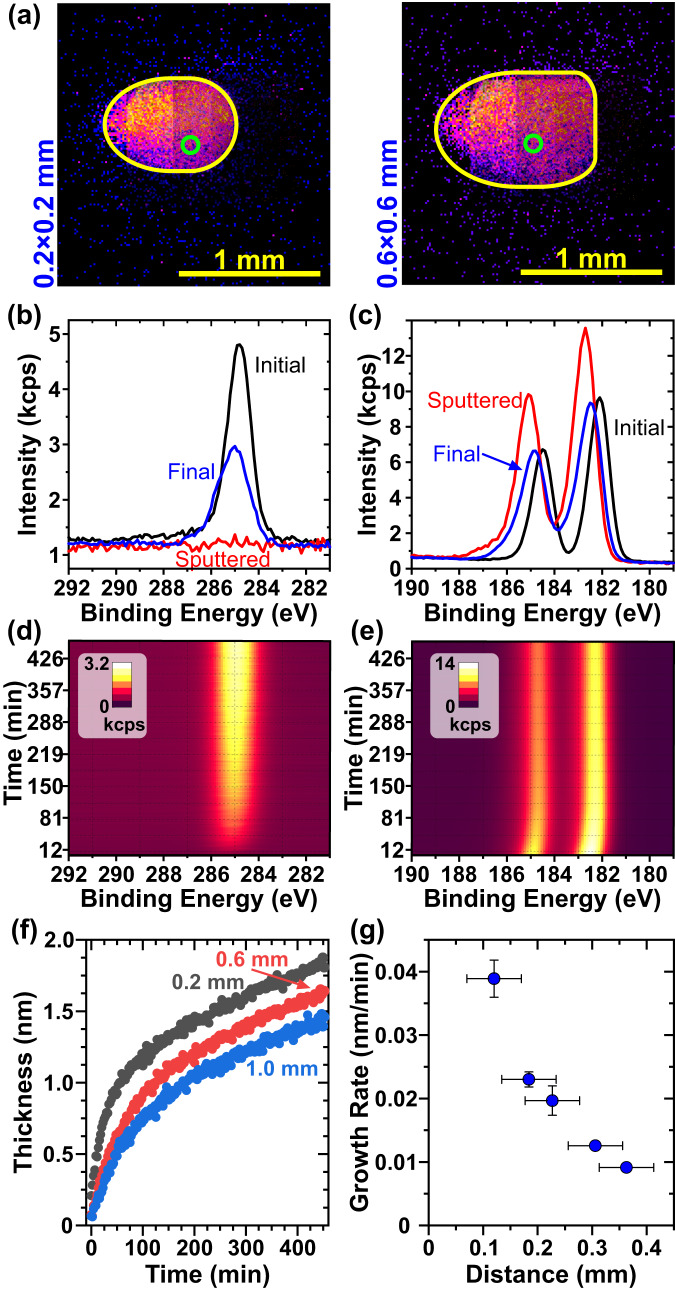
XPS analysis of isoprene flow on zirconia. **a** Before the measurement, the selected area of the sample (highlighted in color) was removed by an ion-cluster beam with a variable raster size given by the image in blue (two nominal raster sizes are shown, a complete set is provided in Supplementary Fig. 1). The actual sizes of these areas were obtained on a reference sample comprising a polymeric layer on Si substrate (see methods) by measurement in an imaging mode at the energy of Si 2p peak; the high intensity of Si is associated with areas where polymer film was removed. **b**, **c** The C 1s (**b**) and Zr 3d (**c**) spectra measured before cluster ion beam sputtering, after the sputtering, and at the end of the experiment; data for nominal size 0.6 × 0.6 mm are shown. **d**, **e** Time evolution of C 1s (**d**) and Zr 3d (**e**) shown as a color profile: each line represents a single spectrum with a color-coded intensity. Each measurement cycle (both C 1s and Zr 3d spectra) took 2.3 min. **f** The effective thickness of the isoprene layer as a function of time after the end of sputtering for three distinct nominal sizes of sputtered area: 0.2 × 0.2 mm, 0.6 × 0.6 mm, and 1.0 × 1.0 mm. **g** Dependence of isoprene height growth rate (in nm min^−1^) on the shortest distance to the void rim. The *x*-error bar represents the size of the analyzed area and the *y*-error one standard deviation of slope of linear fit of the initial isoprene layer thickness increase.

The sample comprising a thin layer of isoprene on the zirconia surface was introduced to the vacuum environment of the XPS instrument through a load-lock chamber. To determine the stability and surface mobility of isoprene molecular layers on the oxide surface, we have removed isoprene from a selected area on the zirconia surface by 2.5 keV ${{{{{{\rm{Ar}}}}}}}_{500}^{+}$ cluster beam (Fig. 1a and Supplementary Fig. 1) and employed XPS to measure the rate of its refilling by diffusion/flow from neighboring areas by monitoring the increase of the C 1s peak intensity as a function of time. The C 1s and Zr 3d peaks measured on the as-prepared sample before sputtering (Supplementary Fig. 2), immediately after the sputtering, and at the end of the experiment (after ~ 7.5 h) are shown in Fig. 1b and c; the color-coded temporal evolution of these peaks is given in Fig. 1d and e. The initial isoprene layer thickness of (2.8 ± 0.5) nm was determined by calculating the ratio of intensities of C 1s/Zr 3d photoelectron peaks in SESSA^52525252^ (see methods). The isoprene thickness did not significantly change during experiments, i.e., during 50 h. ${{{{{{\rm{Ar}}}}}}}_{500}^{+}$ cluster beam efficiently removes the isoprene layer from a given area; however, the C 1s signal quickly increases in time, which is accompanied by a decrease in Zr 3d peak as the substrate photoelectrons are attenuated in the growing isoprene layer. The intensity of C 1s peak reached after 4 h corresponds to (1.5–1.8) nm, which is about half of the initial thickness. The time evolution of equivalent thickness for distinct sizes of sputtered areas is given in Fig. 1f. The measurement shows two regimes: up to ~0.6 nm, the thickness rapidly increases, followed by its steady rise with a lower slope. The limiting thickness is close to the average diameter of the molecule of 0.68 nm, calculated from the molecular mass and isoprene density in a liquid state. The growth rate also depends on the distance from the edge of the sputtered area. The further the measurement area from the edge, the slower the rate at which the isoprene layer grows in thickness, as shown in Fig. 1g. The late steady increase of (0.0014 nm min^−1^) is independent of the distance from the edge. The analogous experiments were performed with isopropanol (Supplementary Fig. 3). Here, the initial intensity increase of 0.004 nm min^−1^ lowered to 0.0012 nm min^−1^ in a steady state growth regime; both values were independent of the sizes of the sputtered area. The initial growth rate for isopropanol was smaller than isoprene, but the steady-state rates were similar for both molecules; these values are at the level observed for adsorption from the residual atmosphere (see below).

To obtain a rough estimate of the speed of molecular flow, we have divided the distance of the measurement area from the edge by the time necessary to form a full monolayer. For all the sputtered areas, it gives a molecular flow of (1.0 ± 0.2) μm s^−1^. To get a more precise estimate, we employ a model in which we consider the diffusion of isoprene from a line source (periphery of the sputtered area). We do not consider adsorption from the residual atmosphere and desorption of molecules to play a significant role. We have measured the adsorption within the XPS system within a period of its heavy use to get a top boundary of hydrocarbon adsorption (see Supplementary Fig. 4). We have determined the initial effective hydrocarbon layer thickness increase to be 0.0013 nm min^−1^, which is below 10% of the thickness increase measured for isoprene. Desorption of molecules during the experiment (and consequent loss of material in a given area) also cannot be ruled out. During the course of the experiment (50 h), we did not observe a significant change in the thickness of the organic layer outside the sputtered areas. Hence, we infer that also desorption has a minor effect on the resulting isoprene thickness. Moreover, the possible desorption goes against the adsorption lowering the impact of both contributions; hence in the following calculation, we do not include both adsorption and desorption; the maximum error will be 10% of provided value, which is well with the confidence interval given below.

By excluding adsorption and desorption, the model reduces to solving a 2D diffusion equation with the following boundary condition: at the edge of the sputtered areas, the layer is one monolayer thick (0.68 nm) and remains constant in time. Thickness measured right after Ar cluster sputtering was taken as the initial value in the entire sputtered area, not considering the profile of Ar beam and potential variations in sputter efficiency. The numerical solution of the 2D diffusion equation was obtained using the finite-difference method using the Python programming language. The diffusion coefficient was estimated by comparing the measured and calculated thickness evolution and minimizing the sum of squared errors. The resulting diffusion coefficient is (40 ± 30) μm^2^/s. This value lies in the range of diffusion coefficients previously determined for systems comprising organic molecules on oxide surfaces. Computation study of *para*-hexaphenyl on $(10\bar{1}0)$ zinc oxide surface determined the value of 5 μm^2^ s^−1^ at room temperature; the fluorination of the molecule with 2 of 4F atoms decreased the diffusion coefficient value to 1 μm^2^ s^−1^ or 0.1 μm^2^ s^−1^, respectively^51^. In another computational study, the diffusion coefficient decreased from 190–0.02 μm^2^ s^−1^ with increasing chain size from benzene to *para*-hexaphenyl on silica surface^43^. An experimental study on the diffusion of stearic acid on the (0001) plane of the water-free surface of α-alumina gave a value^52^ of 20 μm^2^ s^−1^.

### Immobilization of VOC on the ceramic surface

**Fig. 2:**
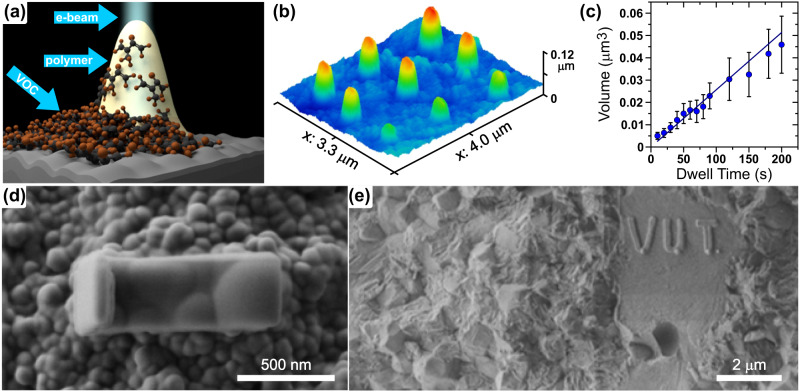
Electron beam writing of nanostructures from VOC. **a** Schematic of VOC (isoprene) polymerization by e-beam. **b** AFM image of the statically grown structures with increasing dwell time. Accelerating voltage 2 kV, beam current 12 pA, dwell times 10, 20, … 90 s, substrate TiO_2_ flat and polished. **c** Dependence of the volume of e-beam written nanostructure on irradiation dwell time. The error bars represent 95% confidence interval. **d** SEM image of the dynamically grown structure. Acceleration voltage 1 kV, beam current 15 pA, substrate fractured nano-3Y-TZP, the real-time video is provided as Supplementary Video SV1. **e** SEM image of dynamically grown structures VUT. Acceleration voltage 1 kV, beam current 15 pA.

Polymerization requires material and energy. We have already determined that the surface flow of synthetic VOCs can provide enough material for localized polymerization. In this section, we will describe the immobilization of VOCs by an e-beam to achieve high molecular weight immobile polymers. Figure 2a demonstrates the expected mechanism of 3D nanostructure formation by polymerizing the organic layer of adsorbed VOCs transferred to the area exposed by the e-beam. Zirconia and titania were used for the substrate preparation; sintering conditions determine the density of polycrystalline materials; for the immobilization studies, we tested substrates with up to 20% porosity with no significant impact of the porosity on the VOCs immobilization. Both smooth and rough surfaces were prepared by fracturing or polishing the sintered materials. Two types of VOC layers were employed. The first one was the isoprene layer deposited as the liquid on the cleaned surface; the liquid evaporated within seconds, and the prepared sample was placed into the closed box. The second kind of sample comprises a natural VOC layer from ambient conditions; according to the previous study, the deposited layer should be similar regardless of a specific indoor environment^19^.

The energy required for polymerization is provided locally via an electron beam. The electrons capable of exciting and ionizing molecules have energies in the range of 5–15 eV. Electrons with energies in this range are produced as a result of the interaction of high-energy electrons with the solid or liquid (secondary electrons)^53^. The interaction volume of primary electrons increases with increasing primary beam energy. To keep the interaction volume low and polymerization localized, the primary e-beam should have low energy on the level of a few keV to penetrate only a small volume of the substrate^53^. We have applied the low accelerating voltage of 0.5–5 kV, beam current 1–1000 pA, and dwell times from seconds to 72 h in SEM conditions with the aim of preparing 3D polymeric nanostructures from VOC present on the ceramic substrate. We have tested both static and dynamic growth. In the static one, a single point was irradiated by a 10 nm beam for a given exposure dwell time (Fig. 2b). In the dynamic growth, the desired structure was written by moving the e-beam (Fig. 2c and Supplementary Video SV1).

An example of an e-beam written structure is given in Fig. 2b, d, e. The longer the e-beam irradiation, the higher and wider the structures, as shown in Fig. 2b. The volume of written nanostructures given in Fig. 2c was calculated from the measured structure height and full width at half maximum considering their conical shape (detailed in methods). In the case of the short-time exposure (10–200 s), the nanostructure volume linearly increases in time with the rate (2.6 ± 0.2) × 10^5^ nm^3^ s^−1^. The linear increase is consistent with earlier studies on electron beam growth employing focused electron beam^31,33,36,38^. The structure presented in Fig. 2d was grown by the dynamic e-beam (see the attached video SV1) during 420 s; in this case, the average volume growth rate was ~7.5 × 10^5^ nm^3^ s^−1^. Importantly, the structure also grows linearly in height, as shown in Fig. 2c, so the material can be supplied to the top of the already-grown polymer. Considering the volume of the single isoprene molecule of 0.17 nm^-3^, we can estimate the flux of monomers for the polymerization by e-beam on the level of millions of molecules per second. The nanostructures can be written in any desired shape (Fig. 2e) and also on rough plus porous substrates (Supplementary Fig. 5).

**Fig. 3:**
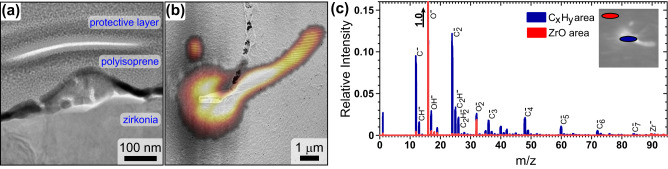
Analysis of prepared nanostructures. **a** TEM cross-sectional view of static e-beam written nanostructure of a conical shape on zirconia substrate. **b** TOF-SIMS map taken at *m*/*z* of C^-^ superimposed on SEM image of polymeric nanostructure prepared by e-beam irradiation (acceleration voltage 2 kV, current 6.6 pA, total time 52,800 s) on zirconia surface. The designed structure comprises a set of 100 nm-spaced points forming three 2000 nm long lines positioned 300 nm from each other. The drift in the later stage of exposure caused the formation of a “comet-like” tail of lines. The intensity of carbon increases from black to white. **c** Mass spectrum measured within and outside of the grown nanostructures. The complete spectra are given in Supporting Figs. 6 and 7.

The cross-sectional TEM image of the statically written nanostructure given in Fig. 3a shows that the nanostructures have approximately a conical shape. Their interior is not compact but comprises nanometer-size pores. The chemical composition of nanostructures was probed by TOF-SIMS (Fig. 3b, c). For this analysis, we have prepared nanostructures with a large volume suitable for the TOF-SIMS analysis by an 18-hour-long exposition of the zirconia substrate to the e-beam radiation. As shown in Fig. 3b, the designed array of rectangles was deformed by charging the non-conductive zirconia surface; in this way, an array of objects with increasing volume up to a few cubic micrometers built from VOC was obtained. In addition to elements contained in the substrate (Zr, O), the mass spectra show only the presence of hydrocarbons. Figure 3c shows the hydrocarbon fragments containing up to 7C atoms and the full spectra given in Supplementary Figs. 6 and 7 fragments up to 16C atoms within the measured range up to *m*/*z* of 200. The measured fragments have a much larger molecular weight than isoprene (C_5_H_8_), confirming the formation of crosslinked polymers by e-beam irradiation. Figure 3c also shows that the large hydrocarbon fragments were detected only on the position of deposited nanostructures.

## Discussion

The isoprene layer on ceramic surfaces behaves as a fluid film with a thickness of around four molecules (2.8 ± 0.2 nm). The VOC multilayer is also stable under ultrahigh vacuum conditions (*p* ~ 10^−6 ^Pa), which indicates relatively strong, liquid-like intermolecular interactions within the layer. The VOC layer is propagated on the ceramic surface at high speed, and the high surface roughness and porosity do not present a significant obstacle (Fig. 2d). The behavior of a VOC film on a ceramic surface is similar to liquid with zero contact angle and no gravity limitation when the thickness of the film is constant on one sample at the same conditions. Similarity with the dynamic wetting of solid by liquid can be observed^54^, but further investigation is needed to define proper models. Draining material from the film leads to rapid replenishment in the depleted region, but the resulting film thickness is reduced. The steady supply of the VOC enables the formation of polymeric nanoobjects employing e-beam immobilization.

To estimate the amount of material diffusing to the area irradiated by an e-beam, we use the mean first passage time required for diffusing particles to cross a boundary^55^. The mean exit time $T\left(r\right)$ for a particle staring in a circular area of diameter $D$ from a concentric area with radius $R$ by a random walk is

$$T\left(r\right)=\frac{{R}^{2}-{r}^{2}}{4D},$$

where $D$ is the diffusion coefficient. By replacing the time average with the average per ensemble and reversing the time, we obtain the mean radius $R$ of the circular area from which the particles reach the smaller area of radius $r$ as ${R}^{2}=4{DT}+{r}^{2}$. We have determined the diffusion coefficient to be ~40 μm^2^/s. Hence for 1 s irradiation and beam radius of 10 nm, the material for polymerization is gathered from the area of ~500 μm^2^ (radius of ~12 μm). Considering a full monolayer of isoprene, the volumetric flow is over 340 × 10^6^ nm^3^. s^−1^. The observed volume growth rate on the spot during polymerization by e-beam is 0.26 × 10^6^ nm^3^. s^-1^ giving 8 × 10^-4^ (0.08%) probability of reaction considering the same film and polymer density. This is in good agreement with our observation that the nanostructures grew during the whole irradiation on the selected area, which shows the steady flow of reactants.

A variety of ceramic surfaces can be used as substrates—nano-sized zirconia fractured surface (Fig. 2d) or micro-sized fracture surface (Fig. 2e). We have also been successful on very rough surfaces and surfaces of titania. We expect similar properties of VOC films on various oxides or oxide layer-covered materials but with different diffusion coefficients. The porosity of the materials is not an essential limiting factor; a porous zirconia matrix with 20% porosity was successfully used to create nanostructures (see Supplementary Fig. 5); however, the polymerization of VOC on zirconia powder (compressed green body) was not successful. There are several possible reasons for it: the high porosity of electrically non-conductive materials leads to surface charging and electron beam scattering, the diffusion of organic material between the non-sintered particles is very limited, or the e-beam can also penetrate deep inside a highly porous material, causing polymerization within the pores without us noticing it. Concerning the last point, the penetration depth of electrons into ceramic material strongly depends on the primary electron energy (see Supplementary Table 1); at 2 keV, the penetration depth is 30–40 nm, depending on the material. Considering the relatively low density of pressed nanopowder, we can expect penetration of electrons through particles (mean particle size is 80 nm). Furthermore, the path of the electrons is not straight (see Supplementary Fig. 8), largely increasing the interaction volume in which the electrons can cause polymerization on pore walls. However, further investigation is necessary to quantify this phenomenon and define the organic layer flow limits on various surfaces and the organic molecule transport within the porous media.

Natural VOC can be utilized in the same way as synthetic ones. Our experiments showed that polymeric nanostructures also grow on untreated surfaces comprising only native hydrocarbon layers. Hence, no pretreatment is necessary to fabricate nanostructures on a variety of surfaces, with the only limit being access to the e-beam. This offers the possibility to move nanotechnology to natural material sources and on complex 3D macrostructures, create a polymeric film on inorganic surfaces, or disarm the harmful VOCs by their immobilization. On the contrary, concentrating molecules from an organic film of unknown composition into a compact localized object makes chemical analysis feasible.

In conclusion, we have determined the molecular flow of VOC on a variety of ceramic substrates as a source of spatially localized chemical reactions. We have shown that the isoprene multilayers are stable on the ceramic surface under ultrahigh vacuum; the molecules spread over the sample at rates of micrometers per second. The methodology presented here provides essential quantitative insight for the modeling of surface chemical reactions and provides an answer to the question: “How far and how fast can the molecules travel on the surface to react?” We have shown that, on average, all molecules from an area with a radius of 12 μm pass at least once under a nanometer-sized electron beam within one second.

## Methods

### Inorganic substrate

Zirconia - 3 mol% yttria-stabilized tetragonal zirconia (TZ 3Yfrom Tosoh Corp., Japan) and titania powders (P25 Aeroxide, Degussa, Germany) were used as starting powders for processing by preparation of suspensions suitable for slip casting followed by sintering, uniaxial pressing at 20 MPa followed by sintering, and direct shaping by Spark Plasma Sintering technique. The sintering temperatures from 1100 °C to 1500 °C were used to tailor the microstructure and density of the substrates. Only fully sintered ceramic surfaces were used for experiments reported in the main text. The porous substrates were also tested; this fact is explicitly stated at each occurrence. The selected samples were cut and polished at a level of 1 micron to achieve a flat surface or manually broken to get the fracture surface with an area of at least 20 mm^2^.

### Organic layer deposition

The organic adlayer was deposited after the thermal cleaning of zirconia or titania surfaces. The thermal cleaning was done at a temperature of 600 °C or higher in the tubular furnace with a flow of air for at least 2 h. Subsequently, during the cooling, the atmosphere was changed to pure nitrogen; the cleaned substrates were stored in HPLC water. The quality and reproducibility of the procedure were verified by the contact angle measurements using the sessile-drop technique^56^. The contact angle of the zirconia surface decreased from 85–81° to 38–34° after the surface cleaning, indicating minor residual contamination. After drying by the compressed air, the substrates with clean surfaces were exposed to ambient conditions or immediately covered by isoprene; such prepared samples were stored in a closed box. The sample was introduced to a vacuum environment through a load-lock chamber, where the sample was kept for 45 min before inserting it into the main chamber with a base pressure of 2 × 10^−9^ mbar. The processing steps are described in Supplementary Fig. 9.

### XPS analysis

The X-ray photoelectron spectroscopy (XPS) analysis was performed on a Kratos Supra spectrometer using monochromatic Al Kα X-ray radiation with 15 mA emission power. The base pressure of the instrument is 2 × 10^−9^ mbar; during the measurement, the pressure increased to 2 × 10^−8^ mbar. The magnetic lens was used for all the measurements. Typical settings were: pass energy of 160 eV and energy step of 1 eV for survey spectra and pass energy of 20 eV and energy step of 0.1 eV for detailed spectra. The size of the analyzed area was restricted to 110 μm in diameter by an aperture. Charge neutralization was used for all measurements. The slight overcompensation was adjusted during the spectra processing by correcting the energy scale to the C 1s position of 285.0 eV.

The isoprene layer was removed from the selected area by 2.5 keV ${{{{{{\rm{Ar}}}}}}}_{500}^{+}$ clusters (5 eV per atom) for 1800 s. Under these conditions, we did not observe any chemical changes (reduction) in the oxide substrate. The size of the sputtered area was determined on a reference sample comprising poly(lactic-co-glycolic) acid (PLGA) layer on a SiO_2_ substrate. The XPS imaging of the reference sample was carried out in the imaging mode at the energy of Si 2p peak employing pass energy of 160 eV. Theoretical modeling of peak intensities was carried out employing the NIST Database for the Simulation of Electron Spectra for Surface Analysis (SESSA)^57^, version 1.2. The model comprised a bulk oxide layer (ZrO_2_) covered with a uniform hydrocarbon layer with an atomic density of 7.8 × 10^22 ^cm^−3^ determined from isoprene molecular mass and density in the liquid state.

### SIMS, SEM, and AFM analysis

Secondary Ion Mass Spectrometry (IONTOF TOF-SIMS5) was employed for elemental analysis of e-beam fabricated nanostructures. We used the instrument in the imaging mode that provides enhanced lateral resolution of ~0.8 µm and sufficient mass resolution of ~140 at C_5_ peak. Bi^+^ primary ions with the following parameters were used: impact energy of 30 keV, impact angle of 45°, pulsed primary current of ~3 pA, and raster size 25 µm × 25 µm. Cs^+^ co-sputtering was performed in the non-interlaced regime with a crater size of 120 µm × 120 µm, an impact angle of 45°, and an energy of 500 eV. The base pressure in the analytical chamber was ~5 × 10^−10^ mbar.

SEM and AFM analysis was performed by two separate SEM instruments—Tescan Lyra3 and FEG-SEM Carl Zeiss ULTRAPLUS. The typical procedure conducted by the LYRA3 SEM was that the chamber was cleaned with a plasma decontaminator at a pressure of 50 Pa for 10 min, and the shape and dwell time (electron dose) were controlled by DrawBeam2 software. The parameters for nanostructure formation were the following: accelerating voltage of 0.5–5 kV, current from 1–1000 pA, and dwell time from seconds to 72 h.

The grown structures were analyzed by AFM (Nenovision Litescope) directly inside the SEM chamber. We did not find any significant changes in the shape of the polymer structures that were measured at ambient temperature after two weeks. Akiyama Probe was used for AFM imaging with the specification: cantilever length 310 μm, thickness 3,7 μm, width 30 μm, n-type, highly doped silicon; Tip radius < 15 nm, tip height 28 μm; Constant force 5 N/m; Resonance frequency 40–60 kHz. Dimensions and shapes of structures were analyzed in Gwyddion v2.47^58^.

### Supplementary information

Supplementary information

Description of Additional Supplementary Files

Supplementary Video 1

## Data Availability

The data that support the findings of this study are available in Figshare with the identifier 10.6084/m9.figshare.24125196^59^.
