# Supplementary material for: Superflux of an organic adlayer towards its local reactive immobilization
Source: Commun Chem. 2023 Oct 18;6:225. doi: 10.1038/s42004-023-01020-2 (PMC10584841; doi:10.1038/s42004-023-01020-2)
Supplement: Supplementary file 1 — Supplementary information [file 42004_2023_1020_MOESM1_ESM.pdf]

SUPPLEMENTARY INFORMATION

Superflux of an organic adlayer towards its local reactive immobilization

David Salamon[1], Kristýna Bukvišová[1], Vít Jan[2], Michal Potoček[1], Jan Čechal[1,2]

[1] CEITEC - Central European Institute of Technology, Brno University of Technology,

Purkyňova 123, 612 00, Brno, Czech Republic.

[2] Fakulty of Mechanical Engineering, Brno University of Technology, Technická 2896/2, 616 69, Brno, Czech Republic.

Supplementary Figures

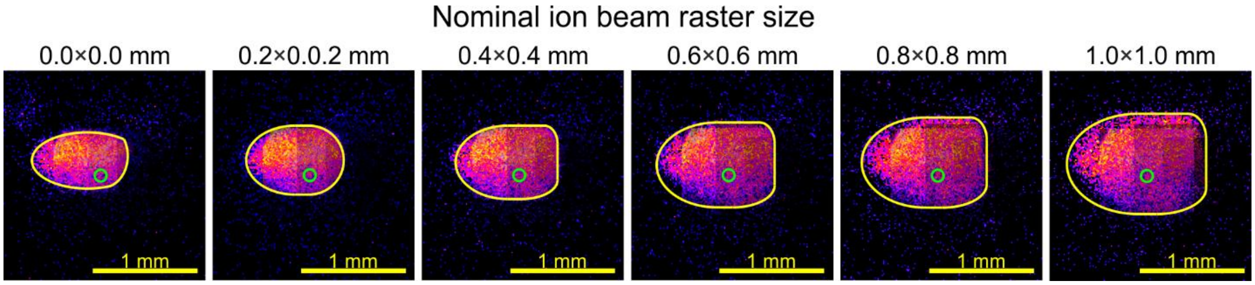

Supplementary Figure 1: (Extended Figure 1a) Measurement of areas where the organic layer is removed by the Ar cluster beam for a set of nominal raster sizes. Photoelectron images at the energy of Si 2p were obtained on a reference sample comprising a polymeric layer on Si substrate (see methods); the high intensity of Si is associated with areas where polymer film was removed.

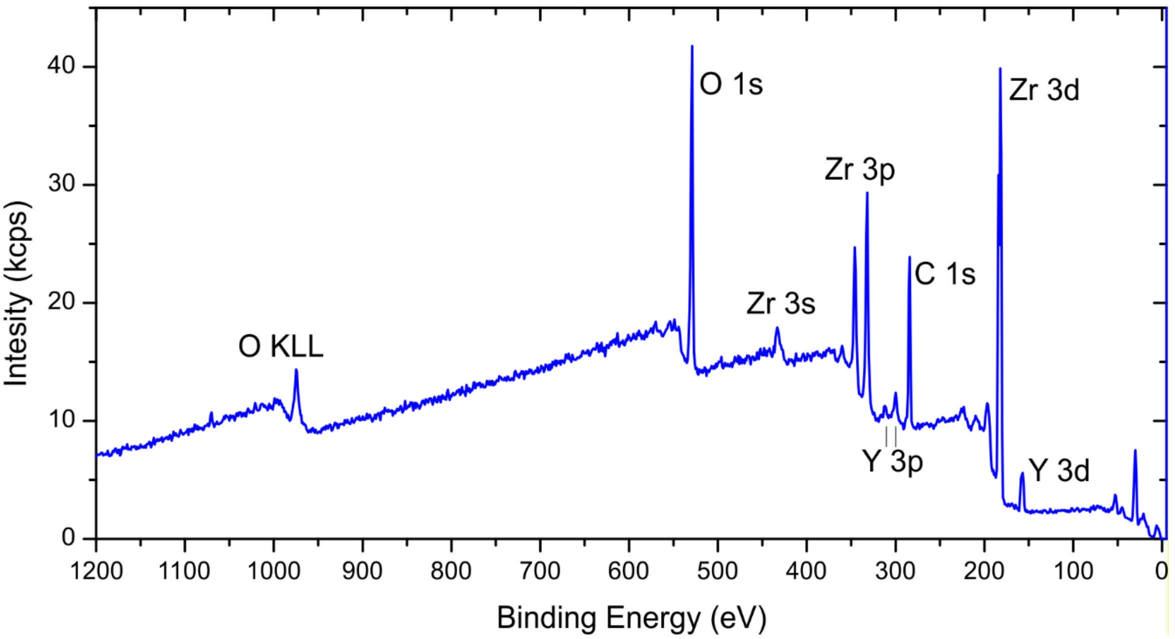

Supplementary Figure 2: Survey spectra measured on an as-prepared sample comprising an isoprene layer on a yttria-stabilized zirconia surface.

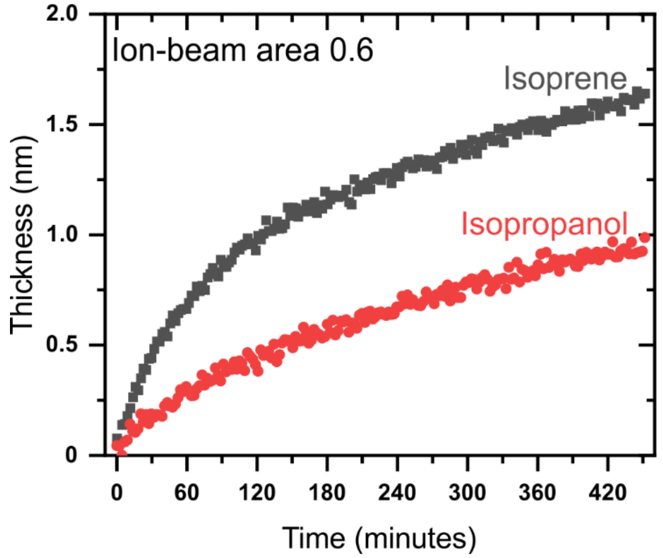

Supplementary Figure 3: Comparison of the effective thicknesses of the isoprene and isopropanol layers as a function of time after the end of sputtering for nominal sizes of the sputtered area of 0.6×0.6 mm.

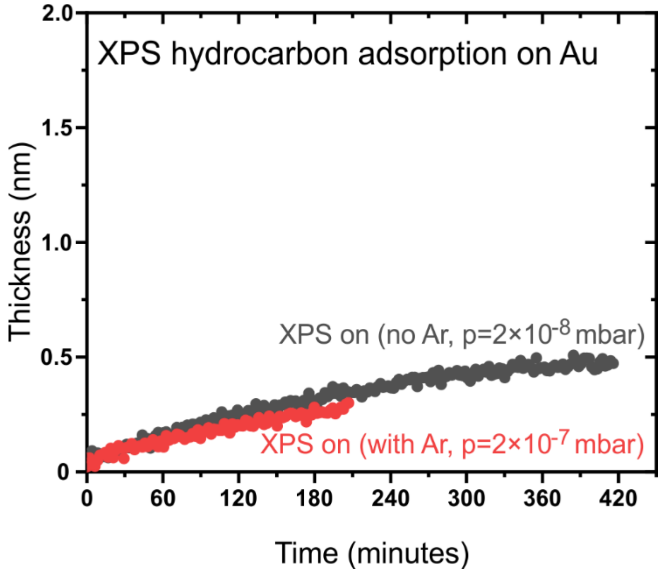

Supplementary Figure 4: Hydrocarbon adsorption from residual atmosphere in XPS instrument. Time evolution of the effective thicknesses of the hydrocarbon layer after the end of monoatomic Ar^+ sputtering (5 keV, 6×6 mm^2, 5 minutes). The black curve was measured when the ion source was turned off immediately after sputtering; for the red curve, the ion source was kept in "standby mode," which resulted in Ar pressure in the chamber (p = 2×10^{-7} mbar). After turning the instrument off, the pressure decreased to 5×10^{-9} mbar in 30 minutes.

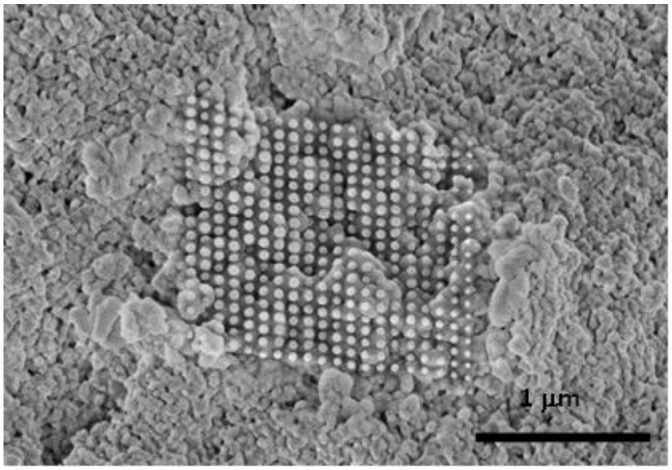

Supplementary Figure 5: The array of immobilized natural VOC by e-beam with accelerating voltage 2kV, beam current 6 pA, dwell time on the spot 5s, and the distance between spots 100 nm. The zirconia substrate was prepared by the SPS technique, and the theoretical density of the sample was around 80 %.

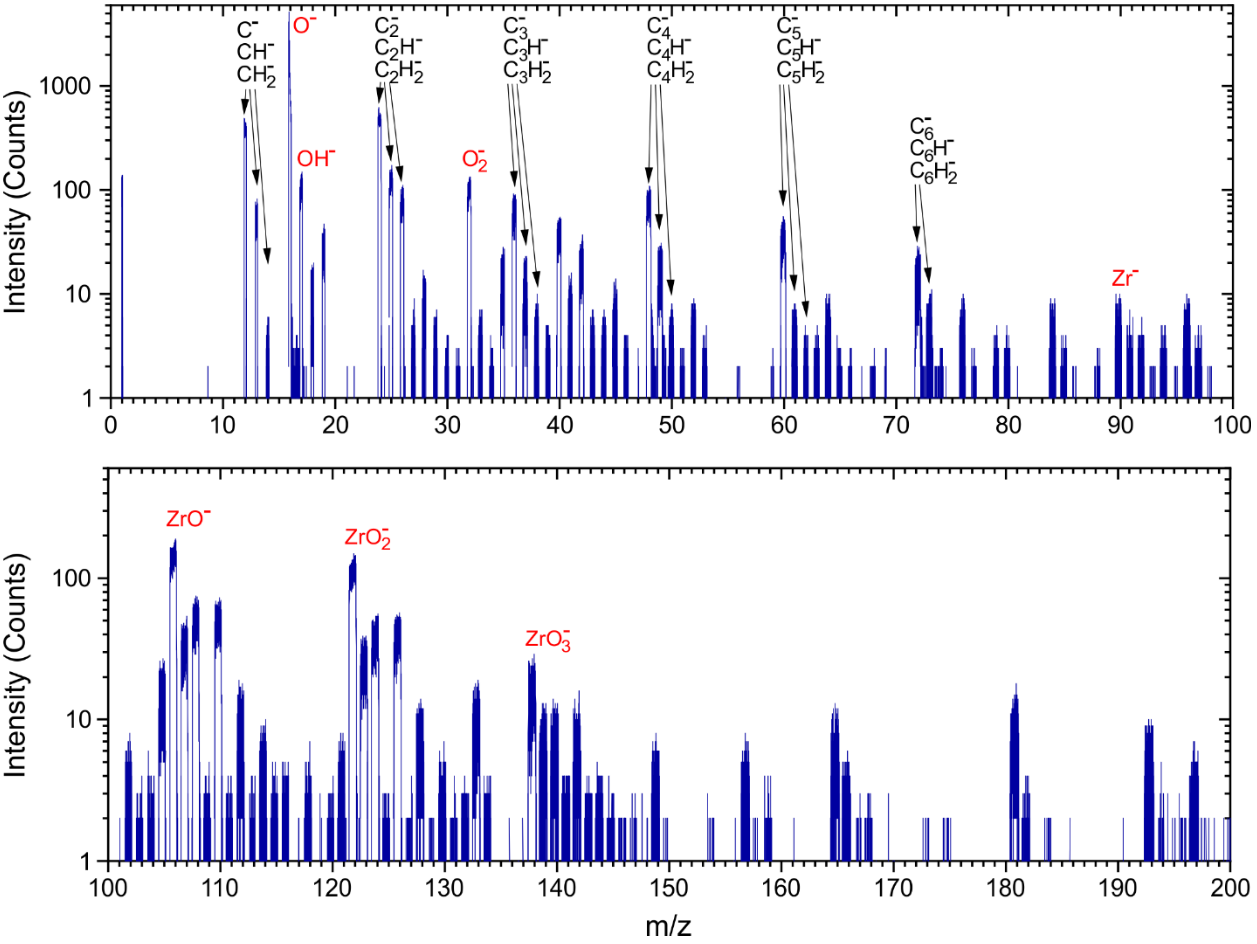

Supplementary Figure 6: Full mass spectrum measured within the grown nanostructures (blue marked area in Figure 3c in the main text.

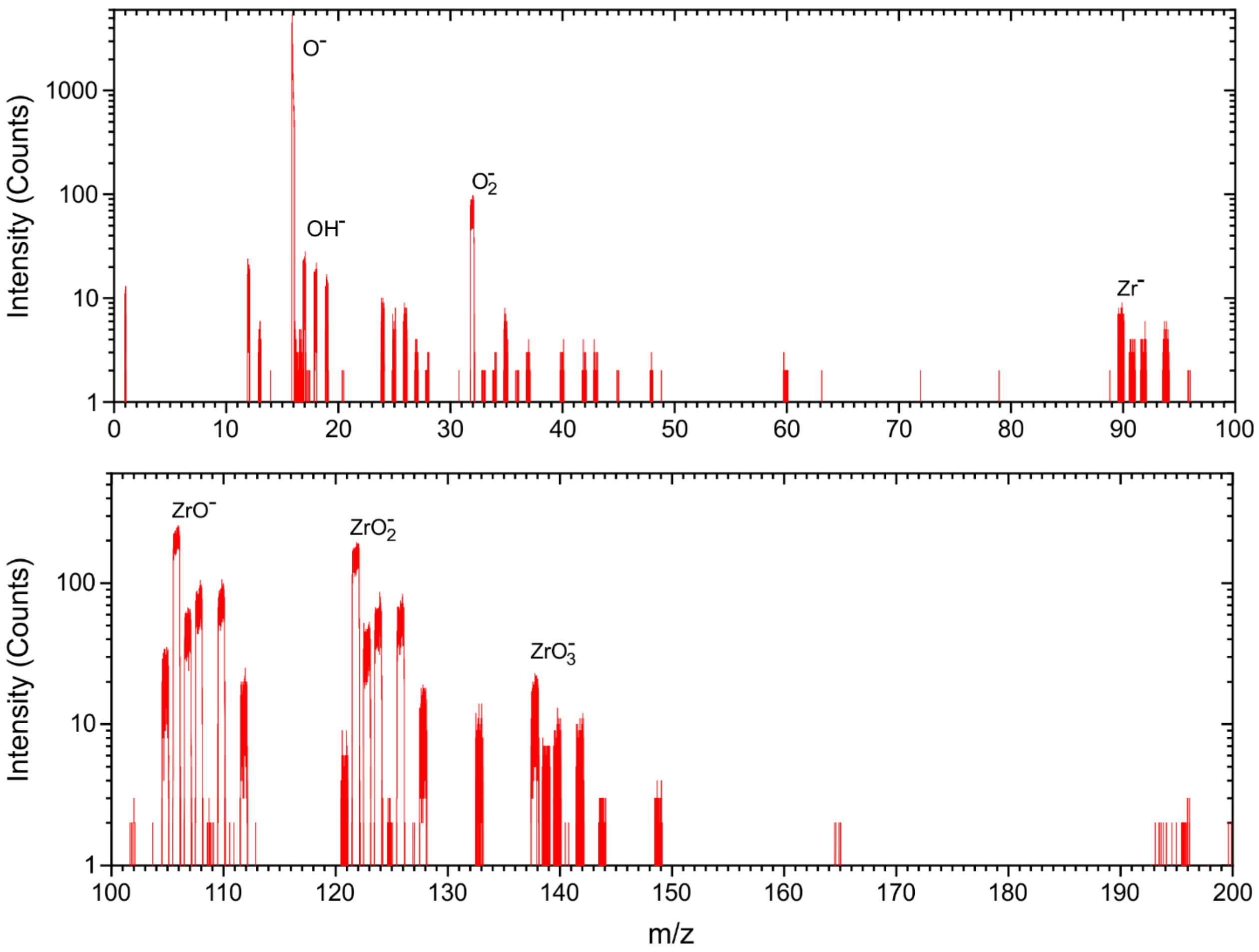

Supplementary Figure 7: Full mass spectrum measured on zirconia surface outside the grown nanostructures, i.e., the red marked area in Figure 3c in the main text.

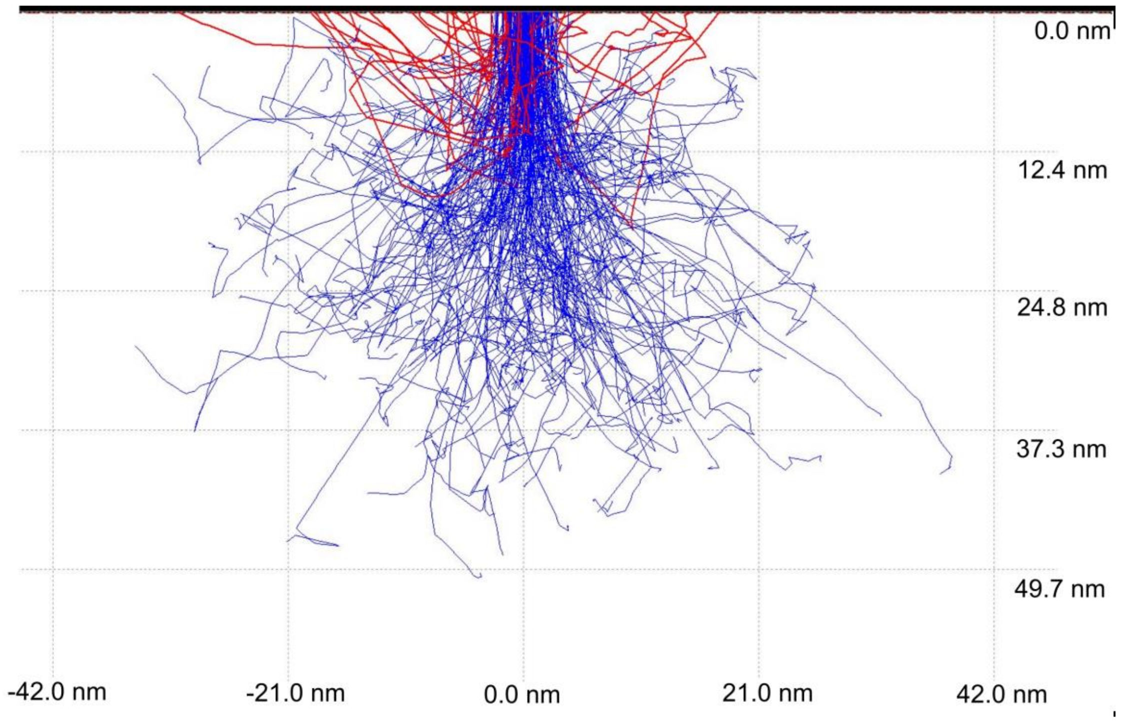

Supplementary Figure 8. Simulation of electron beam penetration in ceramic materials. The simulation of the interaction of low-energy electrons with ceramic material was performed employing the Mott model[1] as implemented in CASINO software v2.42)[2]. The Figure shows the visualization of electron trajectories in titania for 2 kV primary electron energy, spot size 8 nm. Red lines represent electrons that escape from the sample. The penetration depths as a function of primary electron beam energy in dense zirconia (tetragonal zirconia stabilized with 3 mol. % of Y_2O_3) and titania (TiO_2) are given in Supplementary Table 1.

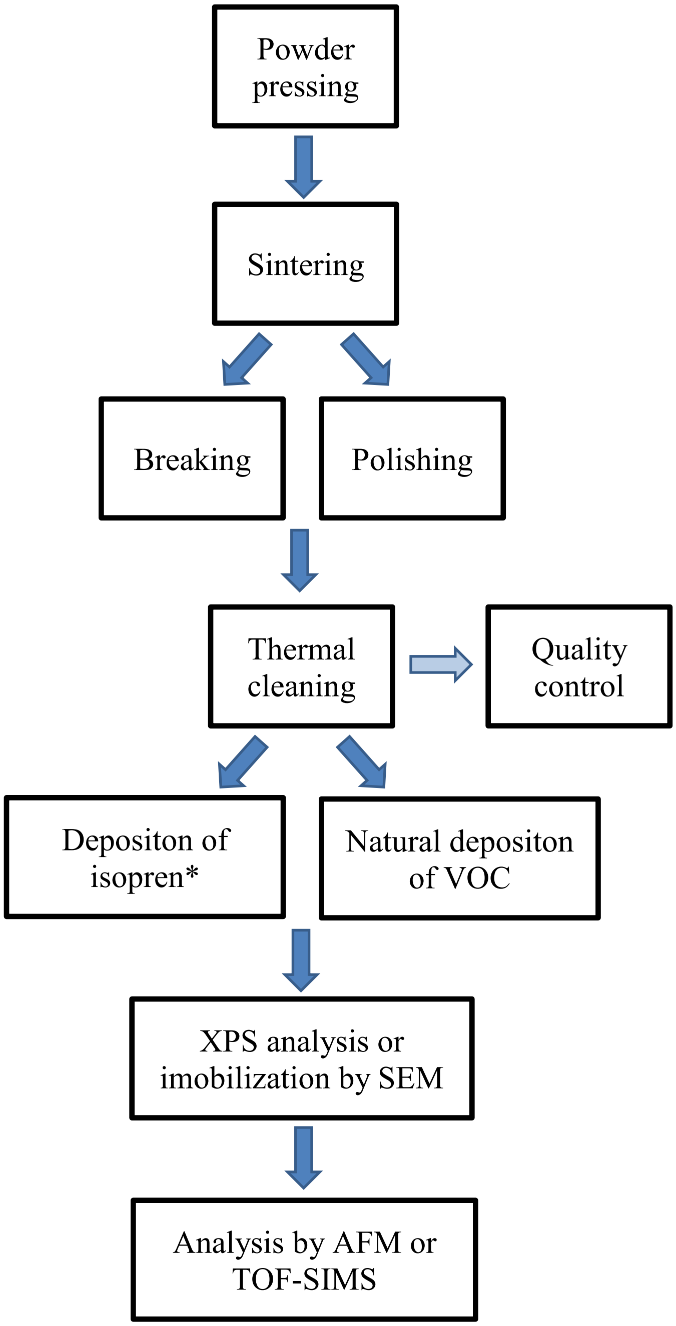

Supplementary Figure 9. Processing steps for sample preparation and analysis. The zirconia and titania were the starting powders. *Note that other synthetic organic compounds like isopropanol were also tested.

Supplementary Tables

Supplementary Table 1. Penetration depths for various primary electron beam energies.

Primary Electron Energy (keV)	penetration depth (nm)	
	titania	zirconia
0.5	6.2	5.5
1.0	15.1	13.5
1.5	26.2	22.7
2.0	38.6	31.8
2.5	54.3	44.5
3.0	70.6	56.1
4.0	112.2	79.5
5.0	152.9	120.0

Supplementary References

1 Bell, D. C. & Erdman, N. Introduction to the Theory and Advantages of Low Voltage Electron Microscopy. *Low Voltage Electron Microscopy: Principles and Applications*, 1-30, doi:10.1002/9781118498514.ch1 (2012).

2 Drouin, D. *et al.* CASINO V2.42 - A fast and easy-to-use modeling tool for scanning electron microscopy and microanalysis users. *Scanning* **29**, 92-101, doi:DOI 10.1002/sca.20000 (2007).
