# Supplementary material for: Superflux of an organic adlayer towards its local reactive immobilization
Source: Commun Chem. 2023 Oct 18;6:225. doi: 10.1038/s42004-023-01020-2 (PMC10584841; doi:10.1038/s42004-023-01020-2)
Supplement: Supplementary file 2 — Description of Additional Supplementary Files [file 42004_2023_1020_MOESM2_ESM.pdf]

Description of Additional Supplementary Files

File name: Supplementary Video 1

Description: Dynamic growth of polymer structure on 3Y-TZP substrate. The electron beam acceleration voltage is 1 kV, and the beam current is 15 pA; the video is recorded in real time.
